# The COVID-19 pandemic from an acute psychiatric perspective: a London psychiatric intensive care unit experience

**DOI:** 10.1192/bjb.2020.54

**Published:** 2020-05-14

**Authors:** Luke Skelton, Ria Pugh, Bethan Harries, Lucy Blake, Margaret Butler, Faisil Sethi

**Affiliations:** South London and Maudsley NHS Foundation Trust, UK

**Keywords:** In-patient treatment, COVID-19, psychiatric intensive care unit, pandemic, comorbidity

## Abstract

The COVID-19 pandemic has put the UK's National Health Service under extreme pressure, and acute psychiatric services have had to rapidly adapt to a new way of working. This editorial describes the experience of a London psychiatric intensive care unit (PICU) where all nine in-patients ultimately tested COVID-19 positive.

The coronavirus disease 2019 (COVID-19) pandemic has put healthcare systems across the world under immense strain, with over 1.2 million cases across 211 countries causing over 70 000 deaths as of 31 March 2020. COVID-19 is a highly infectious novel coronavirus which spreads via droplet transmission, causing a spectrum of disease from mild illness to severe bilateral pneumonia necessitating intensive care treatment.^1^ While acute hospitals prepare for an influx of cases by increasing intensive care capacity and retraining staff, at the time of the events described here there was a lack of specific public health guidance for psychiatric services. In the following weeks, the Royal College of Psychiatrists produced COVID-19 guidance for the acute psychiatric setting and the National Association of Psychiatric Intensive Care and Low Secure Units has published guidance for management of acute disturbance in a psychiatric intensive care unit (PICU) setting.^2^^,^^3^

COVID-19 presents a unique challenge to the in-patient psychiatry setting owing to the nature of psychiatric illness and its treatment. The London PICU described here is a female, 10-bed locked ward comprising two corridors, each with five single bedrooms and two shared bathrooms. There is an extra care area, a seclusion suite, a communal area, including a dining room, and sensory, interview and clinical rooms. At the time of the outbreak, nine of the ten beds were occupied. The tenth bed remained empty over the period described.

Patients who require care in a PICU are in an acutely disturbed phase of a serious mental disorder where the associated risks – to self and others – cannot be safely managed on an acute ward. Their behaviour includes externally or internally directed aggression, unpredictability and vulnerability (overactivity and disinhibition). The PICU model – including ward size, staffing ratio and reduced-stimulus environment – allows for the rapid assessment, management and stabilisation of these patients.

During the COVID-19 outbreak, support and advice were provided by the hospital trust's infection control team. The ward was closed to visitors and staff began wearing full personal protective equipment (PPE) at the point that the first COVID-19 case was suspected. This editorial uses the experience of this London PICU to highlight the challenges that in-patient psychiatric services will face over the coming weeks and offer learning from an evolving situation.

## Timeline of events

In mid-March 2020, case 1 developed a pyrexia of 40.1°C. She had no respiratory symptoms or recent travel history and, owing to the presence of lower urinary tract symptoms, a urinary tract infection was suspected. On day 1 she developed respiratory symptoms and was tested for COVID-19. After developing symptoms, she was encouraged to self-isolate in her room. Over the following 24 h she became increasingly medically unwell, with a deteriorating score on the Modified Early Warning Score (MEWS) assessment and required emergency transfer to an acute hospital. A diagnosis of COVID-19 was confirmed later that day. Table 1 outlines the clinical features and management of the cases on the PICU, with day 0 being the onset of fever for case 1. The median age for the cohort is 34 years (interquartile range: 7.5 years).

**Table 1:** Presenting symptoms, underlying vulnerability factors, adherence to the COVID-19 risk mitigation plan, severity of illness and need for medical transfer in nine infected patients on a psychiatric intensive care unit

Case	Symptom onset	Underlying vulnerability factors	Clinical features	COVID-19 risk mitigation plan	Adherence to isolation	COVID-19 illness severity^a^	Medical transfer required
1	Day 0	PMH: asthma	Pyrexia, cough, tachypnoea >30 breaths per minute, tachycardia, hypotension, saturations<94% on air, reduced GCS	Isolation Intermittent observations and intentional rounding 0.5- to 4-hourly vital signs	Yes	Severe	Yes
2	Day 3	On lithium PMH: obesity, asthma, smoker	Cough, sore throat, coryzal, tachycardia pyrexia	Isolation Intermittent observations and intentional rounding 4-hourly vital signs	No	Mild - Moderate	No
3	Day 4	On ACE inhibitors PMH: hypertension, asthma, obesity. Recent aspiration pneumonia, pulmonary embolism	Pyrexia, shortness of breath, tachypnoea 30 breaths per minute, tachycardia, delirium	Isolation. Intermittent observations and intentional rounding Episodic segregation and seclusion 2- to 4-hourly vital signs	No	Severe	No
4	Day 4	On clozapine and lithium Smoker	Headache, cough, tachycardia	Isolation Intermittent observations and intentional rounding 4-hourly vital signs	No	Mild	No
5	Day 6	On clozapine Smoker	Cough, pyrexia, tachycardia	Isolation Intermittent observations and intentional rounding 4-hourly vital signs	No	Mild	No
6	Day 7	On clozapine and lithium PMH: obesity	Cough, pyrexia, myalgia, tachycardia	Isolation Intermittent observations and intentional rounding 4-hourly vital signs	No	Mild	No
7	Day 7	On lithium Smoker	Pyrexia, sore throat	Isolation Intermittent observations and intentional rounding 4-hourly vital signs	No	Mild	No
8	Asymptomatic Infection confirmed by swab on day 7	Smoker	Pyrexia (one temperature spike recorded in 2 weeks with no other symptoms)	Isolation Intermittent observations and intentional rounding 4 hourly vital signs	No	Mild	No
9	Day 13	On lithium	Cough	Isolation Intermittent observations and intentional rounding 4-hourly MEWS	No	Mild	No

ACE, angiotensin-converting enzyme; MEWS, Modified Early Warning Score assessment; PMH, past medical history.

a. Clinical severity of COVID-19. Mild: mild fever, dry cough, sore throat, headache, myalgia; Moderate: mild symptoms plus shortness of breath; Severe: fever, tachypnoea >30 breaths/min, hypoxia with saturations <94% on air (adapted from Cascella et al^7^).

## The challenges facing acute psychiatric services

The situation in this PICU highlighted some of the challenges faced when managing COVID-19 in an acute psychiatric setting. First, in those with serious mental disorder there is poorer management of physical health than in the general population, particularly comorbidities found to be prognosticators of COVID-19 disease severity: diabetes, hypertension, obesity and smoking.^4^^,^^5^ In addition, this patient cohort is often prescribed psychotropic medication that conveys an independent health risk, such as the cardiovascular and haematological risks associated with clozapine therapy, yet it is not fully understood whether this interacts with COVID-19 pathophysiology.^6^

The management of behavioural disturbance in the context of COVID-19 was particularly challenging because of additional risks, which included: non-adherence to self-isolation procedures and physical health monitoring, the need for interventions such as restraint, rapid tranquillisation and one-to-one nursing. This huge challenge on the PICU contributed to the rapid transmission of COVID-19 between patients. Patients rarely maintained isolation measures and the enclosed layout of psychiatric in-patient wards makes it very difficult to follow social distancing and isolation procedures. There is one clinical room used for medical assessments, investigations and treatment administration. It is difficult to adapt this environment to meet the infection control standards needed to reduce the transmission of COVID-19 while continuing to deliver safe and effective care to patients.

Another challenge is that the mental health nursing workforce undergoes different training and registration from the general nursing workforce, limiting knowledge and experience in managing an acutely medically deteriorating patient. The patients on this PICU presented with a variety of symptoms, many had significantly deranged physical observations and complex comorbidity, yet did not meet the threshold for medical admission during this pandemic and so were treated on the PICU. As a result, their medical management was prioritised while also delivering effective psychiatric care. Many of the staff had little experience of the necessary high-level infection control procedures implemented to manage virus transmission, and this included PPE. This steep learning curve increased staff anxiety levels, potentially further contributing to sickness levels among staff.

Acute psychiatric services will face a number of ethical challenges over the coming months. During this pandemic many patients will be detained to in-patient units under the Mental Health Act 1983 for assessment and/or treatment of their mental disorder. If they become symptomatic for COVID-19 they will be expected to isolate in their room; when this is not adhered to how should it be enforced? Do we need to consider whether they fulfil the capacity criteria to make this decision? A patient's decision no longer affects only them: it also affects other patients, legally detained to the ward, unable to socially distance from the affected individual. The Coronavirus Act 2020 empowers public health officers to authorise initial restrictions to ensure assessment and screening for COVID-19, followed by up to 14 days’ isolation. However, at present it is unclear how this will be implemented. The Mental Health Act allows for a detained individuals’ liberty to be restricted in the context of their mental illness to protect themselves or others, but how should COVID-19 infection affect this? As clinicians and multidisciplinary team members, we are going to have to make difficult ethical decisions using the guidance and frameworks available to us, and our risk assessments must become more complex.

## Summary of learning

Although at the time of writing London is an epicentre for the COVID-19 outbreak in the UK, other acute mental health units will soon be facing similar situations, so it is important to share what has been learnt. All nine patients in this PICU tested positive for COVID-19: two had severe illness, with one requiring transfer to an acute hospital for treatment. One had moderate illness, five had mild illness, and one was asymptomatic at the point of testing but spiked a temperature on day 8. The proportion of severe illness was slightly higher than reported in the emerging global data,^5^ despite the younger age range of the cohort. It is likely that the burden of medical comorbidity among the cohort is the reason for the higher prevalence of severe illness.

As infection control measures were implemented from day 0, with staff utilising full PPE, it was assumed that transmission occurred between patients secondary to the index case. However, this conclusion cannot be made with certainty. As COVID-19 is most infective early in the illness trajectory,^8^ the timeline (Table 1) and the possibility of asymptomatic transmission means that alternative sources and transmission patterns cannot be excluded, reaffirming the need for strict adherence to infection control measures by staff members.

In response to the COVID-19 outbreak the ward had to significantly modify practice. The pace at which new processes were implemented is likely to have been beneficial. Weekly patient-facing ward rounds were suspended and daily multiprofessional meetings were held instead, followed by individual reviews. A risk mitigation plan was developed for each patient; this consisted of a minimum of 4-hourly physical observations, intermittent psychiatric observations plus intentional rounding, and a daily review of their physical status. Increased priority was given to physical health, with daily reviews of physical observations and paracetamol prescription, plus physical examination and phlebotomy where necessary. Before day 4, at which there could be cohorting of COVID-19-positive and -negative patients, medication and meals were taken to the isolating patients. Once cohorted into corridors, a similar process was employed to these distinct areas.

The team liaised closely with the acute medical team to guide supportive management and advise on transfer to the acute hospital. Psychotropic medication was carefully reviewed, with consideration of safe clozapine, lithium and benzodiazepine therapy in the context of COVID-19. Clinical teams could consider reducing the frequency of medicine administration to reduce staff exposure to those with COVID-19. There was close working with the infection control team to quickly implement barrier nursing and upskill ward staff to ensure compliance with public health infection control guidance.

To ensure safe management of acute psychiatric disturbance, the risk assessment was adapted to include COVID-19 infectious status. Encouraging patients to uphold self-isolation was challenging (Table 1). To manage this, the ward was separated into COVID-19-negative and -positive corridors until all patients were confirmed positive. At the point from which the first case was suspected, patients were encouraged to wear masks to minimise transmission, given the poor adherence to self-isolation. Face-to-face assessments were limited to one interaction to prevent repeated staff exposure. Each interaction included all necessary interventions, including mental state assessment, physical examination and phlebotomy. Where physical restraint was indicated, personal protective equipment was worn, including a visor to protect from spitting, and the emergency response team was briefed about the nature of the risks. Zoning, segregation and seclusion were used to manage high-risk behaviours, but not solely as a means of preventing transmission. Where safe to do so, one-to-one nursing took place at the recommended 2 m distance. When rapid tranquillisation was required, consideration was given to the risk of respiratory decompensation in a COVID-19-positive patient.

The guidance and understanding for managing COVID-19 in an acute psychiatric setting is evolving daily, and inevitably will have changed during the timeline of this article. However, the core principles on which clinical teams must base the difficult decisions ahead will remain. The Mental Health Act will remain clear in its remit, but use of the Mental Capacity Act 2005, Coronavirus Act 2020 and common law is likely to increase over the coming weeks when considering how to mitigate the additional and serious risk factors of COVID-19 in our patient cohort.

## About the authors

**Luke Skelton**, MBChB, BSc, MSc, MRCPsych, is a Psychiatry Registrar at South London and Maudsley NHS Foundation Trust, UK. **Ria Pugh**, BSc, BMBS, MRCPsych, is a Core Psychiatry Trainee at South London and Maudsley NHS Foundation Trust. **Bethan Harries**, BA, AKC, BMBS, DMCC, is a Trust Grade Doctor at South London and Maudsley NHS Foundation Trust. **Lucy Blake**, MBChB, MRCPsych, is a Psychiatry Registrar at South London and Maudsley NHS Foundation Trust. **Margaret Butler**, BSc, MSc, MBBS, MRCPsych, is a Core Psychiatry Trainee at South London and Maudsley NHS Foundation Trust. **Faisil Sethi**, MA(Cantab), MBBS, FRCPsych, MScDIC, DipStat, LLM, is a Consultant Psychiatrist at South London and Maudsley NHS Foundation Trust.

## Supplementary material

For supplementary material accompanying this paper visit https://doi.org/10.1192/bjb.2020.54. click here to view supplementary material
